# Design an anthropomorphic dexterous hand for expressive piano performance

**DOI:** 10.3389/fnbot.2026.1775834

**Published:** 2026-04-15

**Authors:** Yanhong Liang, Xianwei Liu, Yanyan Yuan, Chengkai Su, Yucheng Tao, Yongbin Jin, Hongtao Wang

**Affiliations:** 1 Center for X-Mechanics, Zhejiang University, Hangzhou, China; 2 ZJU-Hangzhou Global Scientific and Technological Innovation Center, Zhejiang University, Hangzhou, China

**Keywords:** bio-inspired design, data-driven design, dexterous robotic hand, kinematic analysis, piano performance

## Abstract

**Background:**

Expressive piano performance poses extreme challenges for robotic manipulation, necessitating high-speed repetitive impacts, substantial force output, and coordinated multi-joint control under stringent dynamic constraints. However, existing robotic systems exhibit significant limitations in replicating human-level dexterity, as well as achievable motion speed and force output. This work presents a data-driven, bio-inspired dexterous robotic hand designed specifically for high-fidelity piano performance.

**Methods:**

We first extract kinematic primitives and stable inter-joint coupling patterns from large-scale motion capture data of professional pianists. These human motion priors are directly embedded into the mechanical architecture through morphological coupling and actuator allocation. Actuator selection is further guided by empirically measured human peak velocities and force profiles from biomechanics literature, ensuring sufficient bandwidth for high-speed repetitive motion and adequate force transmission.

**Results:**

Experimental results demonstrate that the proposed hand replicates human-like joint coordination, achieves peak joint velocities of 53.88 rad/s, and provides sufficient fingertip force for authentic piano interaction. As a demonstration of its capabilities, the hand successfully performs a Grade 7 piano piece, Croatian Rhapsody, illustrating its potential for expressive musical performance.

**Conclusion:**

This research establishes a principled pathway from human motion statistics to embodied robotic intelligence, providing a high-performance hardware foundation for autonomous musical performance.

## 1 Introduction

### 1.1 Research background and challenges

Piano performance is widely regarded as one of the most demanding tasks for robotic manipulation (Zakka et al., 2023; Xu et al., 2022). Unlike general grasping (Huang et al., 2025) or industrial assembly, expressive piano playing requires a robotic end-effector to possess a unique combination of high-speed repetitive impacts, wide-range force regulation, and sophisticated multi-joint coordination under stringent dynamic constraints (Hofmann, 1976). To reproduce the touch (le toucher) of a human pianist, the robotic hand must not only cover a vast range of motion to span the keyboard but also achieve high fingertip force and peak angular velocities to produce diverse dynamics from pianissimo to fortissimo.

However, a significant gap remains between human-level virtuosity and the capabilities of existing robotic hardware. Conventional robotic hands often struggle to balance the high power density required for rapid keystrokes with the mechanical dexterity needed for complex fingering techniques. This mismatch limits the ability of robots to perform pieces beyond simple monophonic melodies, hindering the transition from mere mechanical hitting to truly expressive performance.

### 1.2 Evolution of piano-playing robotic hardware

The hardware evolution of piano-playing robots reflects a transition from functional task execution to the pursuit of human-like expressive virtuosity. To bypass the complexity of hand translation, dedicated multi-actuator systems prioritize keyboard coverage over anatomical fidelity. Platforms such as TeoTronico (Music-making piano droid [The Big Picture], 2017) utilize up to 53 independent actuators to control each key directly, excelling in polyphonic complexity and orchestral collaboration.

Parallel to these dedicated systems, the development of low-DOF biomimetic hands focused on basic finger independence and morphology. Early milestones were marked by the WABOT-2 (Sugano and Kato, 1987), which pioneered a 14-DOF configuration for melody playback. Research like (Zhang et al. 2009) and others (Lin et al. 2010) has explored optimized trajectory planning and score-based finger allocation to compensate for restricted mobility. Modern iterations, including commercial platforms like the Inspire-Robots (Inspire Robots, n.d.), BrainCo Revo2 (BrainCo, 2025), and Linker Hand (Linkerbot, 2025), typically provide 1–2 DOF per finger. Despite these advancements, their limited joint mobility often precludes complex lateral movements, such as the thumb-under technique, and fails to achieve the peak velocities required for high-tempo, virtuosic passages.

To address these kinematic limitations, high-DOF research platforms have been employed to validate complex coordination and trajectory optimization in musical tasks. Utilizing advanced commercial hardware such as the Shadow Hand (Shadow Robot Company, n.d.) and Allegro Hand (Wonik Robotics, 2012), researchers have demonstrated sophisticated multi-finger coordination (Scholz, 2019; Zeulner et al., 2025). Despite their dexterity, these high-DOF systems often encounter a significant bottleneck in performance dynamics: they struggle to balance high joint mobility with the peak fingertip output forces and strike velocities characteristic of human masters. Consequently, while they excel at positioning, they frequently fall short of the mechanical power required for fortissimo execution.

The frontier of hardware design now emphasizes the fusion of biomimetic structures and functional materials to overcome the robotic tone of traditional rigid systems. (Hughes et al. 2018) proposed a skeleton-inspired hand featuring passive joints that mimic the kinematic chain of human phalanges, allowing for adaptive posture adjustment. Other anthropomorphic designs (Zhang et al., 2011) have integrated tendon-driven mechanisms to better simulate the human musculoskeletal synergy. Recent advancements (Zhang et al., 2025) have further introduced rigid-flexible hybrid bionic fingers that incorporate compliant materials and sensory components at the joints. These designs aim to achieve variable compliance and energy dissipation during key strikes, enabling refined control over timbre and dynamics through inherent mechanical properties rather than purely algorithmic control.

While current research has significantly advanced kinematic dexterity and structural compliance, expressive piano performance imposes coupled requirements on both finger coordination and dynamic capability.

From a kinematic perspective, achieving expressive piano performance requires a principled trade-off between dexterity and structural simplicity, motivating the identification of appropriate degrees of freedom and dominant hand coordination patterns grounded in the movement characteristics of expert pianists. Prior studies in human hand kinematics and robotic manipulation have shown that, despite the high dimensionality of finger joints, coordinated hand motions can be represented in a low-dimensional space via principal component analysis (PCA) (Jin et al., 2022; Pei et al., 2022). Large-scale analyses of human grasp kinematics have shown that a limited number of principal components explain the majority of variance in daily grasp postures and finger coordination patterns, supporting the synergy hypothesis in motor control and robotic design (Jarque-Bou et al., 2019; Shenoy and Varadhan, 2025). These findings suggest that, rather than arbitrarily increasing degrees of freedom, dexterous robotic hands can be systematically designed by preserving the principal coordination modes observed in skilled human movements.

From a dynamic perspective, expressive piano performance at high technical difficulty imposes stringent requirements on peak torque output and fingertip velocity, which exceed those of most conventional manipulation tasks. Although existing robotic hands can accurately reproduce coordinated postures and motion trajectories, their actuation systems are typically optimized for precision and compliance rather than high power density. As a result, such platforms often fail to generate the rapid force buildup, repeated high-speed impacts, and sustained dynamic loading required for fast keystrokes and forte articulation. This discrepancy between coordinated kinematic control and dynamic capability remains a primary bottleneck in achieving human-level virtuosity in robotic piano performance.

### 1.3 Contributions of this work

To address the limitations of existing systems, this paper proposes a novel data-driven design framework for an expressive piano-playing dexterous hand. The core contributions of our work are as follows:

- Expert-driven kinematic benchmarking: we established a comprehensive metric system for robotic piano playing by performing large-scale motion capture of professional pianists. This data-driven approach quantifies critical design constraints, such as range of motion, joint coupling ratios, and peak angular velocities, thereby providing a physiological blueprint for anthropomorphic hardware.
- Criterion-based mechatronic synthesis: based on the identified kinematic priors and coupling patterns, we implemented a modular, linkage-driven hand with optimized motor selection. This ensures that the hardware's torque and speed capabilities are precisely mapped to the requirements of complex piano repertoire, simplifying the control space without sacrificing dexterity.
- High-dynamic performance validation: through rigorous testing, we verified that our prototype achieves a peak angular velocity of 53.88 rad/s and a peak fingertip force of 33.24 N. The platform's superior power density was demonstrated by the successful performance of the high-difficulty Grade 7 piece, Croatian Rhapsody, confirming its ability to handle the rapid articulation and explosive dynamics required for virtuosic repertoire.

## 2 Kinematic analysis of human pianist motion and configuration of degrees of freedom

To achieve high-fidelity piano performance, the robotic hand must transcend simple mechanical execution and replicate the nuanced coordination of a human pianist. This section details the data-driven framework used to determine the optimal DOF configuration. By analyzing motion capture data from professional pianists through Principal Component Analysis (PCA) and Pearson correlation, we identify the critical joint synergies and kinematic priorities that guide our bio-inspired hardware design.

### 2.1 Experimental setup and data acquisition

**Figure 1:**
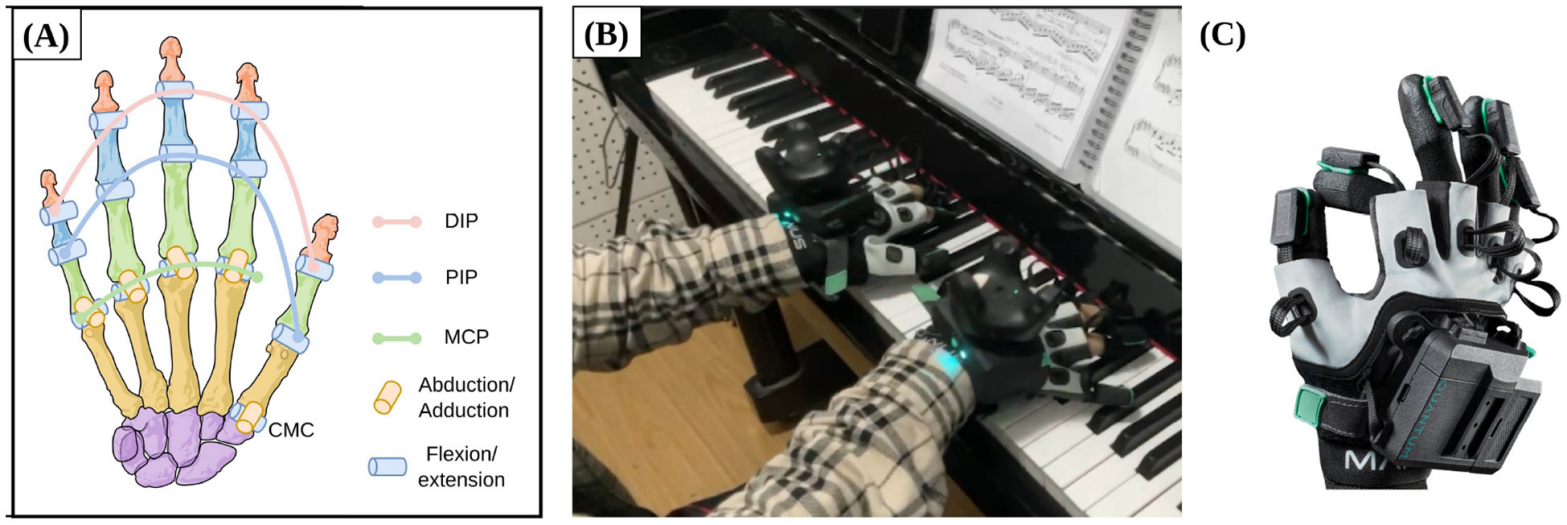
Experimental environment and data acquisition system. **(A)** Schematic of the 20 tracked finger joint DOFs; **(B)** Experimental scene of a professional pianist performing on an acoustic piano; **(C)** The MANUS Quantum Metagloves used for high-precision motion capture.

The human hand is a sophisticated biomechanical system comprising 27 bones and over 20 DOFs. For this study, we focus on the primary joints involved in piano playing: the Metacarpophalangeal (MCP), Proximal Interphalangeal (PIP), Distal Interphalangeal (DIP) joints for flexion/extension, and the Abduction/Adduction (ABD) for lateral movement. To accurately represent the hand's kinematic workspace, we define and track a total of 20 critical degrees of freedom (DOFs), as illustrated in the joint schematic in Figure 1A. Among these, the thumb is treated as a specialized case involving the multi-axial Carpometacarpal (CMC) joint to account for its unique role in complex fingering techniques.

To record high-fidelity motion data without hindering the pianist's natural expression, we employed MANUS Quantum Metagloves (Group, 2024) (Figure 1C). These gloves utilize advanced magnetic-shielded inertial sensing technology, providing a high sampling frequency of up to 120 Hz. Such specifications are critical for capturing the transient dynamics of piano playing, including rapid repetitive keystrokes and subtle staccato articulations. The experimental scene, where a professional pianist performs on a standard acoustic piano while equipped with the motion capture system, is depicted in Figure 1B.

This setup allows for the precise recording of rapid keystrokes, staccato articulations, and complex fingering transitions. All data were collected in a quiet piano practice room using a standard acoustic piano to minimize environmental interference. A professional pianist participated in the experiment. Prior to formal recording, the pianist was given sufficient time to practice while wearing the motion-capture gloves to ensure natural performance without discomfort or movement restriction. Only recordings in which the musical pieces were performed fluently and without noticeable execution errors were retained for analysis.

Five representative musical excerpts covering a broad range of technical demands were selected:

- Twinkle Twinkle Little Star (duration: 00:00:42.983), including basic rhythmic variations and staccato articulation;
- Für Elise (duration: 00:01:50.133), featuring moderately increased tempo and expressive phrasing;
- Czerny Etude Op. 718 (00:01:34.133), designed to train finger dexterity and containing frequent chordal structures;
- An excerpt from Bach's Well-Tempered Clavier (00:01:27.150), representing the fastest piece among the five and characterized by dense polyphonic passages;
- Arabesque (duration: 00:01:46.367), involving significant lateral hand movements and imposing higher demands on finger abduction/adduction control.

Together, these pieces span a spectrum from elementary articulation patterns to high-speed polyphonic execution and lateral workspace utilization, providing a relative comprehensive dataset that captures diverse kinematic requirements of piano performance.

### 2.2 Principal component analysis (PCA) and joint dominance

To reduce the dimensionality of the 20-DOF motion space and identify the core movement primitives, we performed PCA on the standardized joint angle time-series data.

As shown in Table 1, the first principal component (PC1) accounts for a significant portion of the total variance for the index, middle, and ring fingers, with values typically exceeding 75% (reaching as high as 87.9% for the middle finger). This suggests that their flexion movements are primarily dominated by a single synergistic pattern, or a one-dimensional manifold in the joint space. While the PC1 contribution for the pinky finger is lower at 61.5%, it remains the dominant factor compared to higher-order components, indicating a structured movement pattern. In stark contrast, the thumb exhibits a more distributed variance across PC1, PC2, and PC3 (44.3%, 30.7%, and 25.0%, respectively). This indicates that the thumb possesses a lower degree of kinematic coupling and requires more independent active control to execute specialized roles such as Thumb-under maneuvers and complex positioning.

**Table 1:** Portion of total variance accounted for by the first three principal components.

**Finger**	**PC1**	**PC2**	**PC3**
Thumb	0.443	0.307	0.250
Index	0.753	0.207	0.034
Middle	0.879	0.101	0.016
Ring	0.825	0.149	0.024
Pinky	0.615	0.347	0.038

To further understand the composition of these movements, we analyzed the PC1 loadings to identify the dominant joint—the joint that contributes most significantly to the primary motion primitive. The results across different musical repertoires are detailed in Table 2.

**Table 2:** Dominant joint degree of freedom (DOF) for PC1 across different repertoires.

**Repertoire**	**Thumb**	**Index**	**Middle**	**Ring**	**Pinky**
Midrule Twinkle Twinkle	ABD	DIP	MCP	PIP	PIP
Für Elise	CMC	PIP	PIP	PIP	MCP
Czerny Op. 718	ABD	MCP	MCP	PIP	PIP
The Well-Tempered Clavier	ABD	PIP	MCP	PIP	PIP
Arabesque	CMC	PIP	PIP	PIP	PIP

This demonstrates that while the first principal component (PC1) serves as the primary driver of finger motion, the specific joint that dominates this component is not constant but varies across different musical repertoires. Given that instances where the DIP joint acts as the dominant DOF are remarkably rare across the dataset, it is most reasonable to prioritize the MCP, PIP, and ABD joints as independent active DOFs when determining the degree-of-freedom configuration. This selection ensures that the robotic hand retains the necessary kinematic flexibility to accommodate the diverse joint-level priorities and technical complexities inherent in professional piano performance.

### 2.3 Correlation analysis and joint synergy

**Figure 2:**
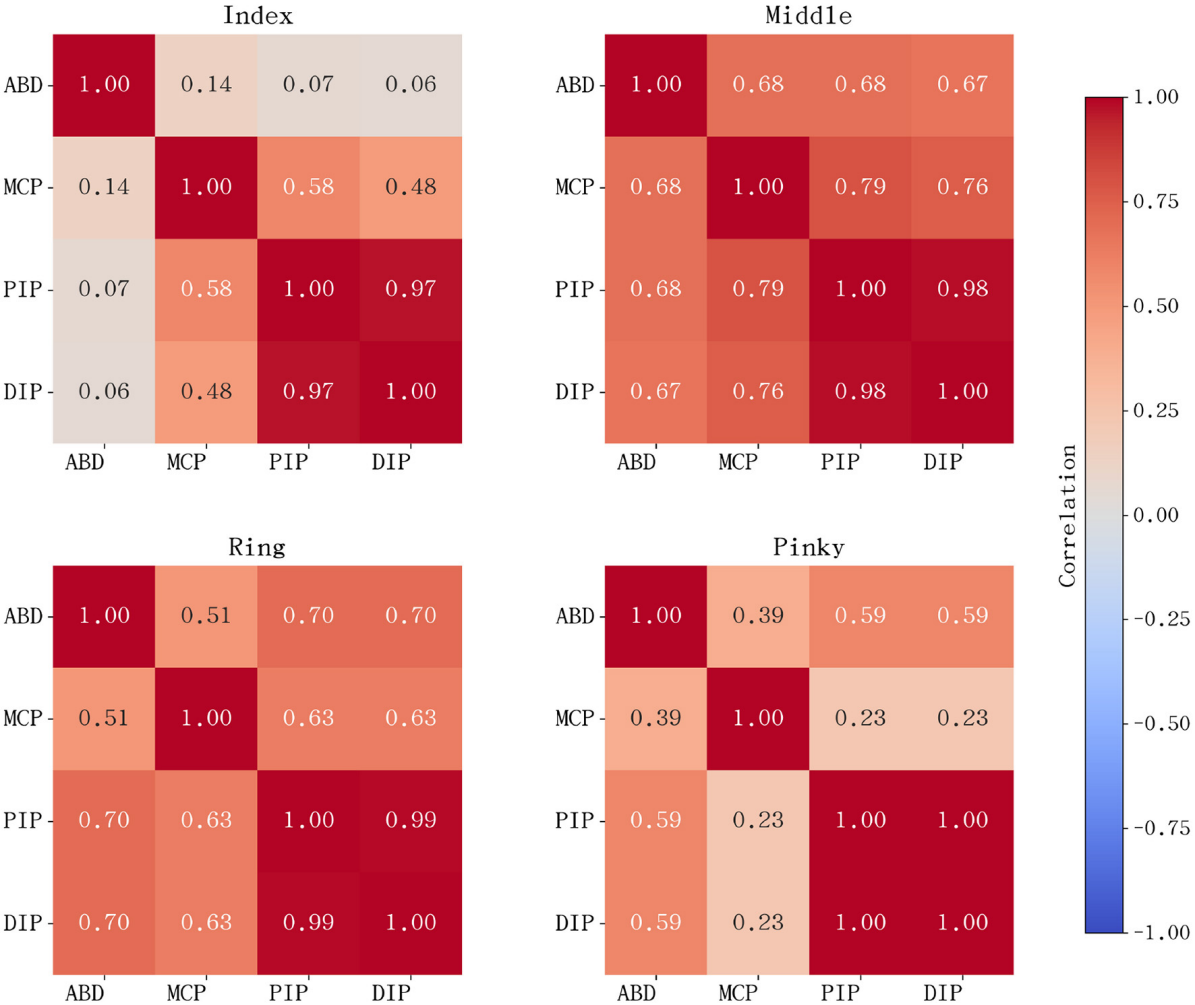
Pearson correlation heatmaps for the primary four fingers (index to pinky). The matrices represent the correlation coefficients between MCP, PIP, DIP, and ABD joints, highlighting the consistent synergistic patterns across different technical maneuvers.

While PCA identifies global motion structures, Pearson correlation analysis provides a granular view of the local linear relationships between specific joint pairs. This analysis is critical for identifying potential joint couplings that can simplify the mechanical design without sacrificing biomimetic fidelity. The correlation analysis was performed on joint kinematic data aggregated across all five musical pieces. Figure 2 illustrates the correlation heatmaps for the index, middle, ring, and pinky fingers.

The most salient finding is the near-perfect positive correlation (*r*≈1.0) between the PIP and DIP joints, which remains remarkably stable across all musical pieces and performance speeds. This robust synergy indicates that the distal part of the finger operates as a highly coordinated functional unit during the attack and release phases of a keystroke. Consequently, this supports the mechanical implementation of a coupled PIP-DIP mechanism. Furthermore, moderate-to-high correlations were observed between the ABD and flexion joints (MCP/PIP) in specific fingers, particularly during large interval jumps or complex chordal transitions. This suggests that lateral positioning is often synchronized with the vertical striking motion to optimize the finger's reach and impact angle.

**Figure 3:**
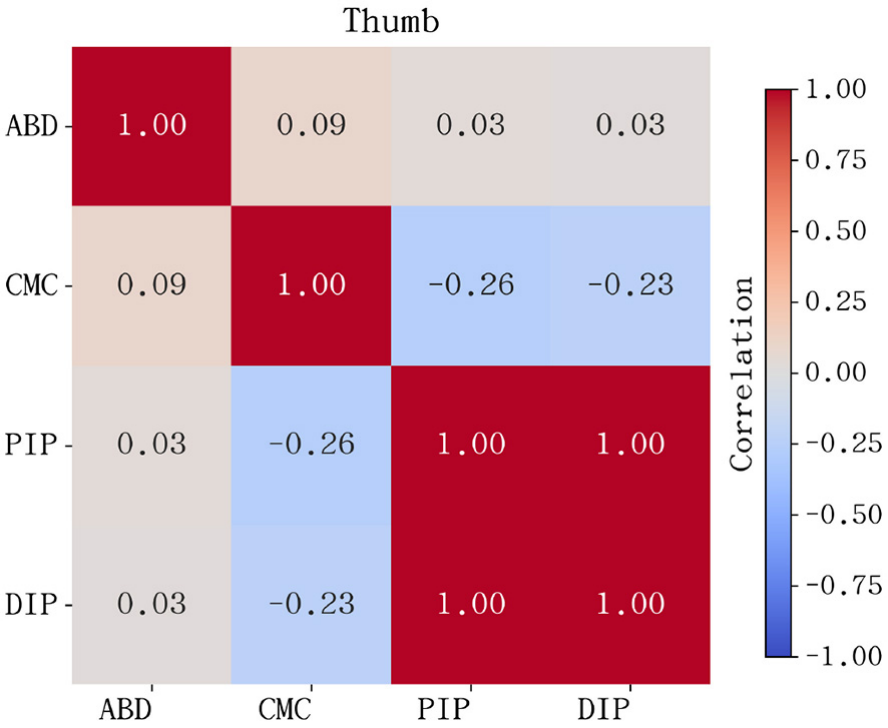
Pearson correlation heatmap for the thumb joints. The distinct patterns, particularly involving the CMC joint, reflect the thumb's unique multi-axial mobility and its relative independence from the distal joint synergies seen in the other four fingers.

For the thumb, the correlation patterns are distinct from the other four fingers, as shown in Figure 3. Although a degree of coupling exists between its distal segments, the CMC joint exhibits a low or even slightly negative correlation with the flexion joints. This statistical independence confirms the CMC joint's role in autonomous posture adjustment and Thumb-under maneuvers. These findings further justify the requirement for higher independent degrees of freedom in the thumb's mechanical design to accommodate its unique functional contributions to piano performance.

### 2.4 Quantitative modeling of DIP-PIP coupling and DOF allocation

The robust joint synergies identified in Section 2.3 provide a biomechanical justification for reducing the control complexity of the robotic hand. To translate these biological observations into a mechanical design, we quantitatively modeled the relationship between the PIP and DIP joints.

By projecting the high-dimensional joint trajectories onto the PC1 axis—which represents the primary flexion-extension synergy—we extracted the dominant motion manifold. A linear regression model was then applied to characterize this synergy (Jin et al., 2022):


$$\begin{array}{l} \theta_{\text{DIP}}=k\cdot \theta_{\text{PIP}}+b \end{array}$$ (1)

where *k* represents the coupling coefficient and *b* denotes the angular offset. This modeling approach ensures that the resulting mechanical coupling captures the maximum variance of natural human movement during piano playing.

**Figure 4:**
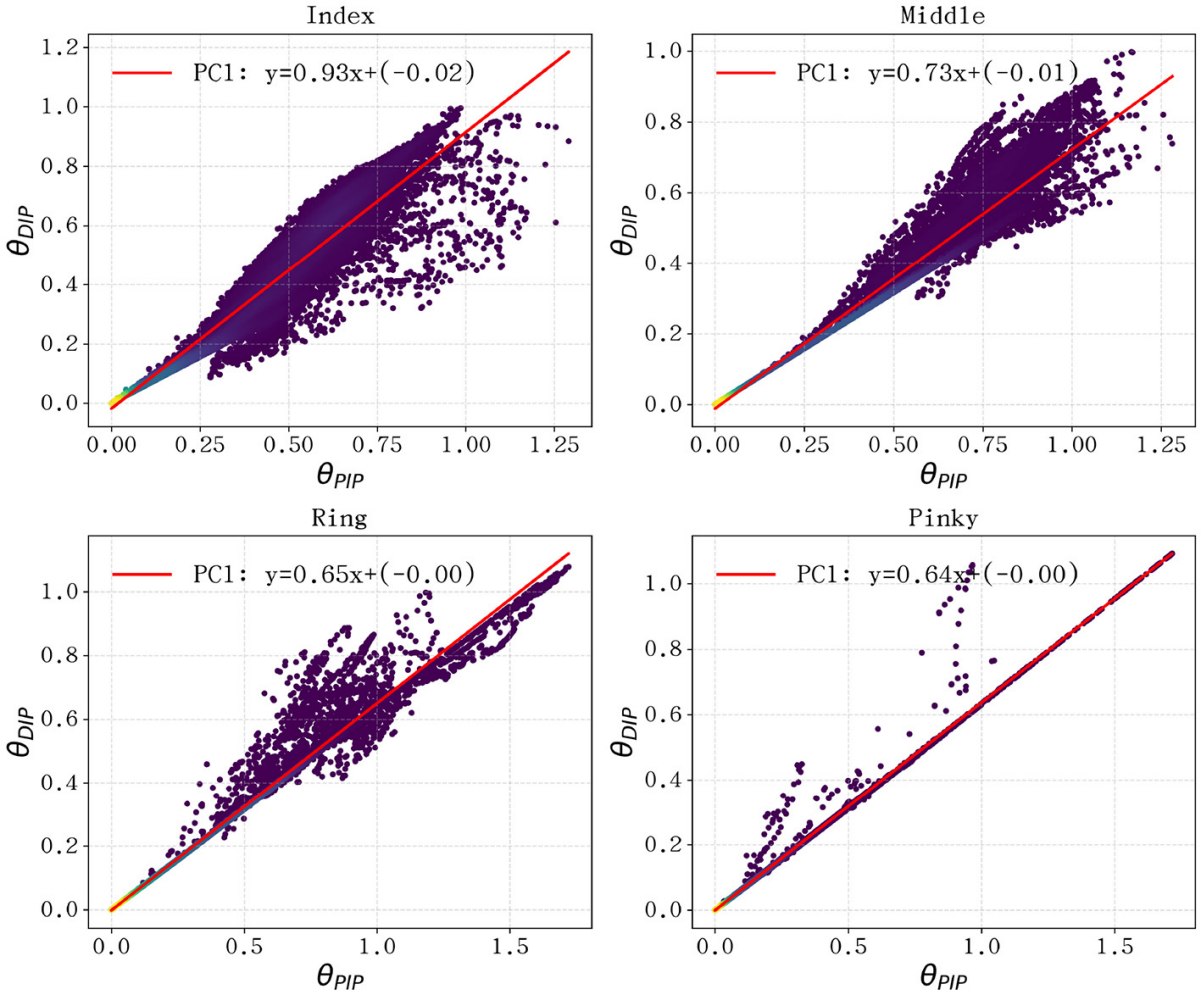
Linear coupling analysis of the DIP-PIP joints for the index, middle, ring, and pinky fingers. The scattered points represent joint angle trajectories projected onto the PC1, demonstrating a consistent linear synergy across different performance scenarios.

**Figure 5:**
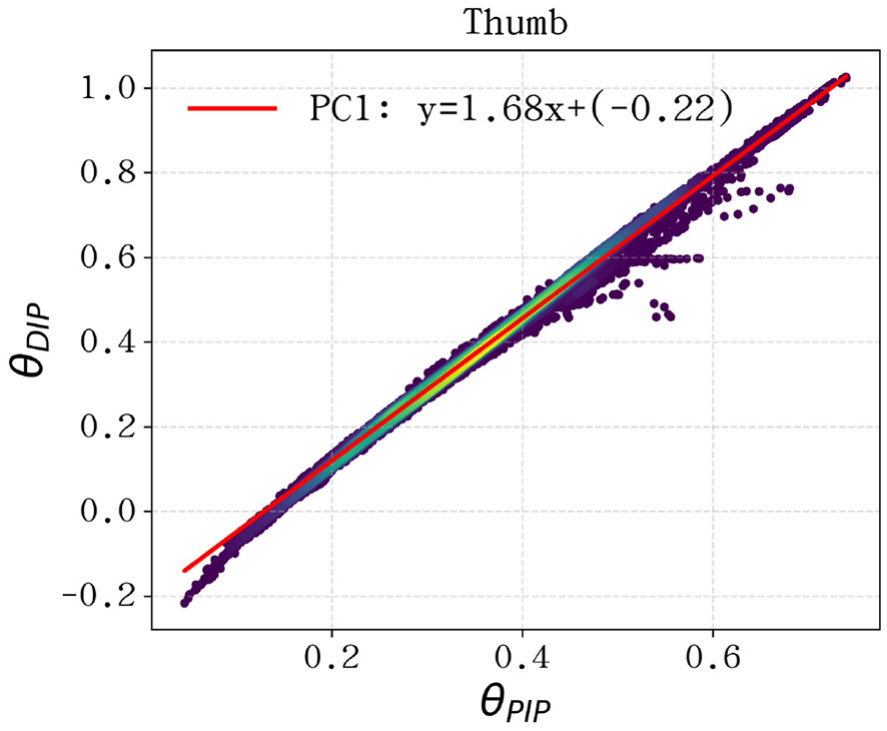
DIP-PIP joint coupling analysis for the thumb. Despite the thumb's unique multi-axial mobility, the distal segments maintain a structured linear relationship suitable for mechanical simplification.

The discrete distribution of joint angles and their corresponding linear fits are illustrated in Figure 4 for the index, middle, ring, and pinky fingers, and in Figure 5 for the thumb. For the four primary fingers, the data points cluster tightly around the regression line, confirming that the PIP-DIP coordination remains highly linear throughout the entire workspace required for musical performance.

The calculated coupling coefficients (*k*) for each finger across all five repertoires are summarized in Table 3. The results reveal a remarkable consistency for the middle, ring, and pinky fingers, with mean coefficients of 0.756, 0.692, and 0.638, respectively. While the thumb and index fingers exhibit higher individual coefficients (1.85 and 0.96, respectively), we opted for a unified mechanical coupling ratio of *k*≈0.7 for the DIP joints of all five fingers.

**Table 3:** Linear coupling coefficients *k* for PIP and DIP joints across selected five repertoires.

**Repertoire**	**Thumb**	**Index**	**Middle**	**Ring**	**Pinky**
Midrule Twinkle Twinkle	1.94	1.04	0.79	0.75	0.63
Für Elise	1.65	1.00	0.74	0.66	0.63
Czerny Op. 718	1.99	0.84	0.75	0.62	0.63
The Well-Tempered Clavier	1.97	0.95	0.69	0.66	0.63
Arabesque	1.70	0.99	0.81	0.77	0.67
Mean	**1.85**	**0.964**	**0.756**	**0.692**	**0.638**

The bold values indicate the mean values.

This design decision balances biomimetic accuracy with engineering practicality. While our empirical data suggests a distinct ratio of *k* = 1.85 for the thumb, implementing a standardized *k* = 0.7 across all fingers facilitates a modular transmission system and simplifies the manufacturing process. By allocating the remaining three DOFs (MCP, PIP, and ABD) as active joints, the hand retains the versatility to execute task-specific priorities identified via PCA, while the passive DIP coupling replicates the human hand's physiological under-actuation. Although future iterations should incorporate a specialized thumb module to further enhance naturalism, the current unified design provides a robust and streamlined hardware foundation.

### 2.5 Final synthesis of the DOF strategy

Based on the aforementioned statistical evidence, we established the following DOF configuration strategy for the robotic hand:

- Three active DOFs per finger: the MCP (flexion), PIP (flexion), and ABD (lateral) joints are configured as active DOFs. This ensures that the hand can replicate the dominant movement modes identified in PCA and provides the lateral flexibility required for complex repertoire.
- One coupled DOF per finger: the DIP joint is implemented as a passive, mechanically coupled joint driven by the PIP joint with a fixed ratio of *k* = 0.7. This reduces the actuator count and control complexity while maintaining a human-like kinematic envelope.
- Modular architecture: to streamline engineering design, all five fingers utilize a modular three-active and one-coupled structure. This provides the thumb with the necessary independence for multi-axis movement while optimizing the overall power-to-weight ratio of the dexterous hand.

## 3 Analysis of dynamic velocity and force requirements for task-driven dexterous hand

The expressive nature of piano performance stems from the intricate modulation of keystroke velocity and impact force. Achieving human-level virtuosity requires the robotic hand to possess high-frequency response capabilities and sufficient power density. This chapter quantifies the dynamic limits of human pianists through time-frequency analysis and establishes a force transmission model to map fingertip requirements to actuator specifications, providing a rigorous foundation for motor selection.

### 3.1 Spatiotemporal and frequency characteristics of human pianists

To ensure that the robotic system can replicate the rapid transients and nuanced dynamics of professional performance, we analyzed the motion of a pianist performing Bach's Well-Tempered Clavier. This repertoire was selected specifically for its high rhythmic density and polyphonic complexity, representing the upper bounds of kinematic demands in our dataset.

#### 3.1.1 Dynamic limits in the time domain

**Figure 6:**
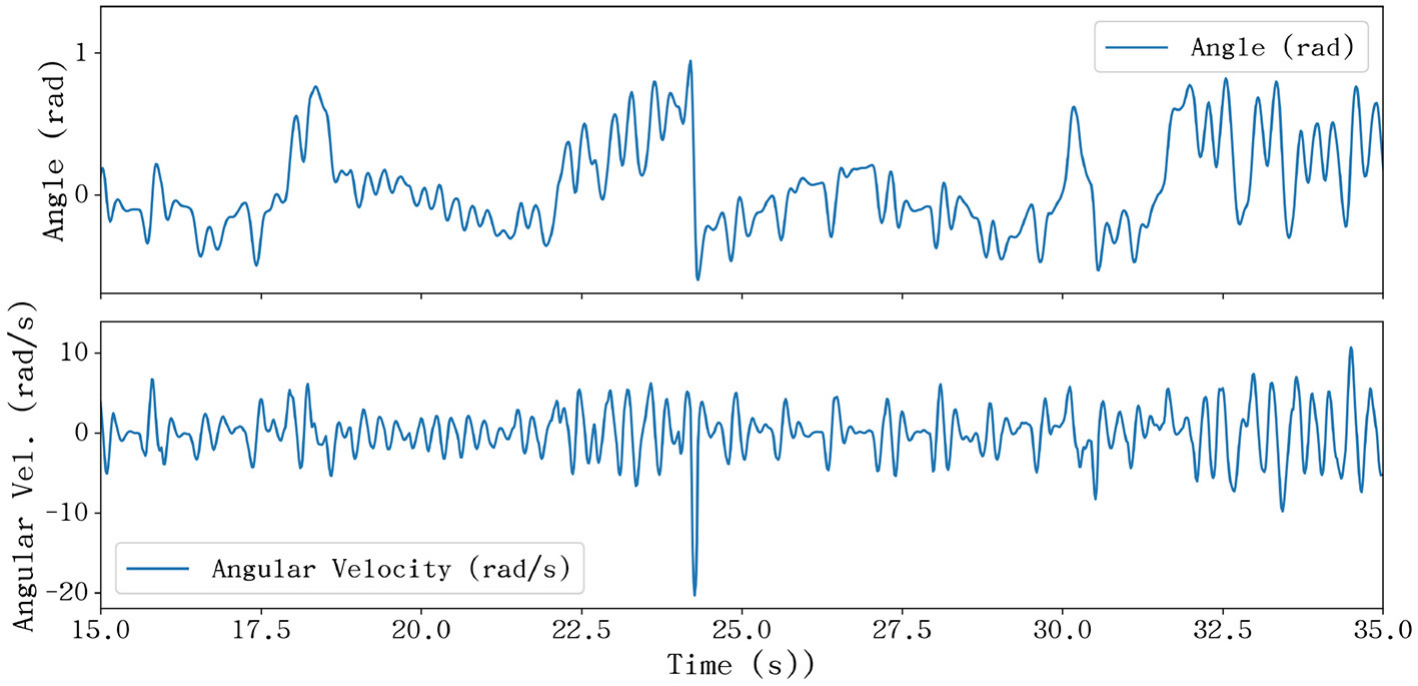
Kinematic profiles of the middle finger MCP joint during Bach's Well-Tempered Clavier. The plots illustrate the joint angle **(top)** and angular velocity **(bottom)**.

The peak hardware requirements were determined by identifying the most demanding trajectories in the dataset, such as the rapid scales in Bach's Well-Tempered Clavier. Figure 6 showcases the angular profile of the middle finger's MCP joint, which exhibits the maximum angular velocity among all joints. Additional joint trajectories are available in Supplementary Figure S1.

During the instant of peak angular velocity, a distinct decrease in the joint angle is observed. The detailed spatiotemporal evolution of this motion, including synchronized video frames, simulation snapshots, and joint angle trajectories, is illustrated in Supplementary Figure S2. This indicates that the maximum angular velocity is achieved while the finger is transitioning from a flexed state to an extended state, a motion characteristic of the rapid release and strike cycle in virtuosic playing. The corresponding angular acceleration supports this observation: the joint undergoes a rapid acceleration phase in the direction of extension, followed by an immediate deceleration upon approaching the target position. At this critical juncture, the MCP joint reaches an instantaneous peak angular velocity of 20.3 rad/s, defining the burst capability required for the actuators to replicate the sharp transients of human articulation. The realization of such dynamic response often necessitates advanced hardware design, as seen in the optimization of soft finger actuators and flexible mechanisms to achieve human-like compliance in robotic systems (Elsamanty et al., 2025).

**Figure 7:**
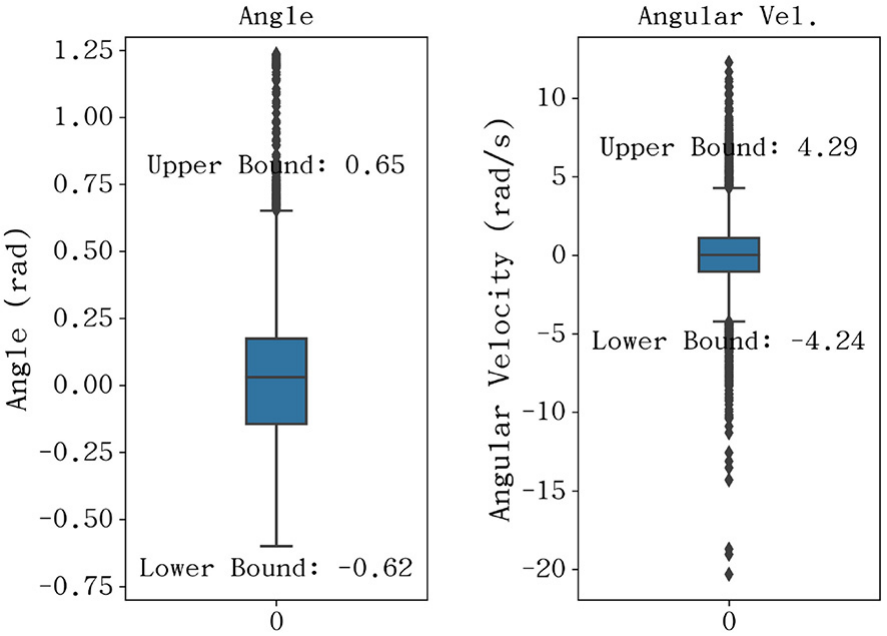
Statistical distribution of joint angle **(Left)** and angular velocitiey **(Right)**.

While the peak kinematic values define the necessary power envelope of the hardware, the statistical distribution of these movements provides a more holistic view of the typical operational requirements. As illustrated in the boxplot analysis (Figure 7), the joint angles are predominantly concentrated within a range of approximately ±0.65 rad. This cluster identifies the core workspace where the robotic hand will spend the majority of its duty cycle, thus necessitating the highest levels of tracking precision and control stability in this specific region. Furthermore, the angular velocity distribution reveals that the bulk of musical articulation occurs within a moderate interval of [−4.24, 4.29] rad/s.

#### 3.1.2 Frequency domain and control bandwidth

To further elucidate the dynamic frequency characteristics of joint movements, we applied the Fast Fourier Transform (FFT) to the recorded joint angle time-series. As an efficient numerical algorithm for mapping time-domain signals into the frequency domain, FFT allows for the quantitative characterization of complex, non-stationary joint motions by decomposing them into a superposition of frequency components. Similar frequency-domain analysis techniques have been widely applied in characterizing biological signals and robotic hand systems to ensure accurate feature extraction and motion identification (Guo et al., 2022; Orban et al., 2019).

From the resulting power spectra, two critical features were extracted: the dominant frequency and the cutoff frequency. The dominant frequency, defined as the frequency component with the maximum amplitude after removing the DC offset, represents the primary rhythm of the joint's periodic motion. The cutoff frequency (*f*_*c*_), which defines the effective bandwidth of the motion, was determined using the cumulative spectral energy method. Specifically, *f*_*c*_ is the frequency at which the integrated energy density *E*(*f*) reaches a threshold of η = 95% of the total energy:


$$\begin{array}{l} \frac{\int_{0}^{f_{c}}E\left(f\right)df}{\int_{0}^{f_{max}}E\left(f\right)df}=0.95 \end{array}$$ (2)

This cutoff frequency captures the maximum range of physically significant high-frequency dynamics, providing essential constraints for the closed-loop bandwidth and dynamic response requirements of the robotic control system.

The spectral distribution across the five fingers (see Supplementary Figure S3 for complete results) indicates that the dominant frequencies of most joints are concentrated within the 0.03–0.18 Hz range. This low-frequency range corresponds to the macro-scale modulation of phrasing and rhythmic cycles in human performance, suggesting that joint angle variations are dominated by slow-moving structural changes rather than high-frequency oscillations. Furthermore, the higher rhythmic modulation observed in the index and middle fingers reflects their functional role in executing rapid melodic passages, which demand higher rhythmic activity compared to the ring finger and pinky.

**Figure 8:**
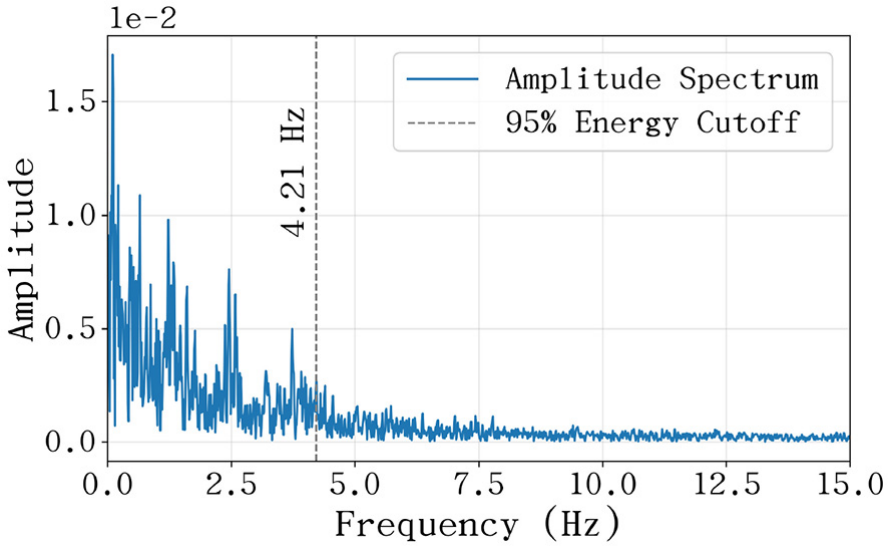
Spectral analysis of pinky MCP joint. The plots identify the dominant rhythmic components and the effective bandwidth required to capture 95% of the signal energy, establishing the baseline for control system performance.

Despite the low-frequency dominance of the rhythms, the effective bandwidth required to capture the rapid transients of keystrokes is significantly higher. The analysis reveals that the effective frequency bands for the joints lie between 1.4*and*4.2 Hz. Notably, the MCP joints consistently exhibit higher cutoff frequencies compared to the PIP and DIP joints. As illustrated in Figure 8, the pinky MCP joint reaches a peak cutoff frequency of 4.21 Hz, representing the upper limit of dynamic modulation in high-speed performance. This suggests that the MCP joints are the primary drivers of high-frequency transients during rapid playing, while the distal joints focus on posture refinement and impact stability.From a control systems perspective, the dominant frequency dictates the steady-state tracking requirements, while the cutoff frequency constrains the minimum dynamic response bandwidth. To ensure that the robotic hand can replicate human-like transients without significant phase lag, the closed-loop bandwidth should ideally be 2–3 times the motion's cutoff frequency. Consequently, a target control bandwidth of 8–12 Hz was established for the joint controllers. This design enables high-fidelity tracking of virtuosic maneuvers while maintaining system stability, adopting a precise control strategy comparable to those employed in high-performance prosthetic systems to ensure stable and intuitive motion (Orban et al., 2020).

### 3.2 Actuation force mapping and transmission analysis

The translation of high-level musical dynamics into precise mechanical execution requires a hardware platform capable of delivering both high-speed articulation and substantial strike force. For this purpose, we adopted a linkage-driven anthropomorphic structure inspired by (Kim et al. 2021). This specific architecture was selected for three primary reasons: first, its configuration of three active DOFs per finger (MCP, ABD, and PIP) aligns with the essential joint priorities identified in our human motion analysis; second, the inherent rigidity of the linkage system ensures high mechanical bandwidth, which is essential for achieving the sharp transients and precise force modulation required for expressive finger-key interaction; and third, the platform offers a mature framework for inverse kinematics, facilitating the derivation of precise mapping between actuator space and task space.

**Figure 9:**
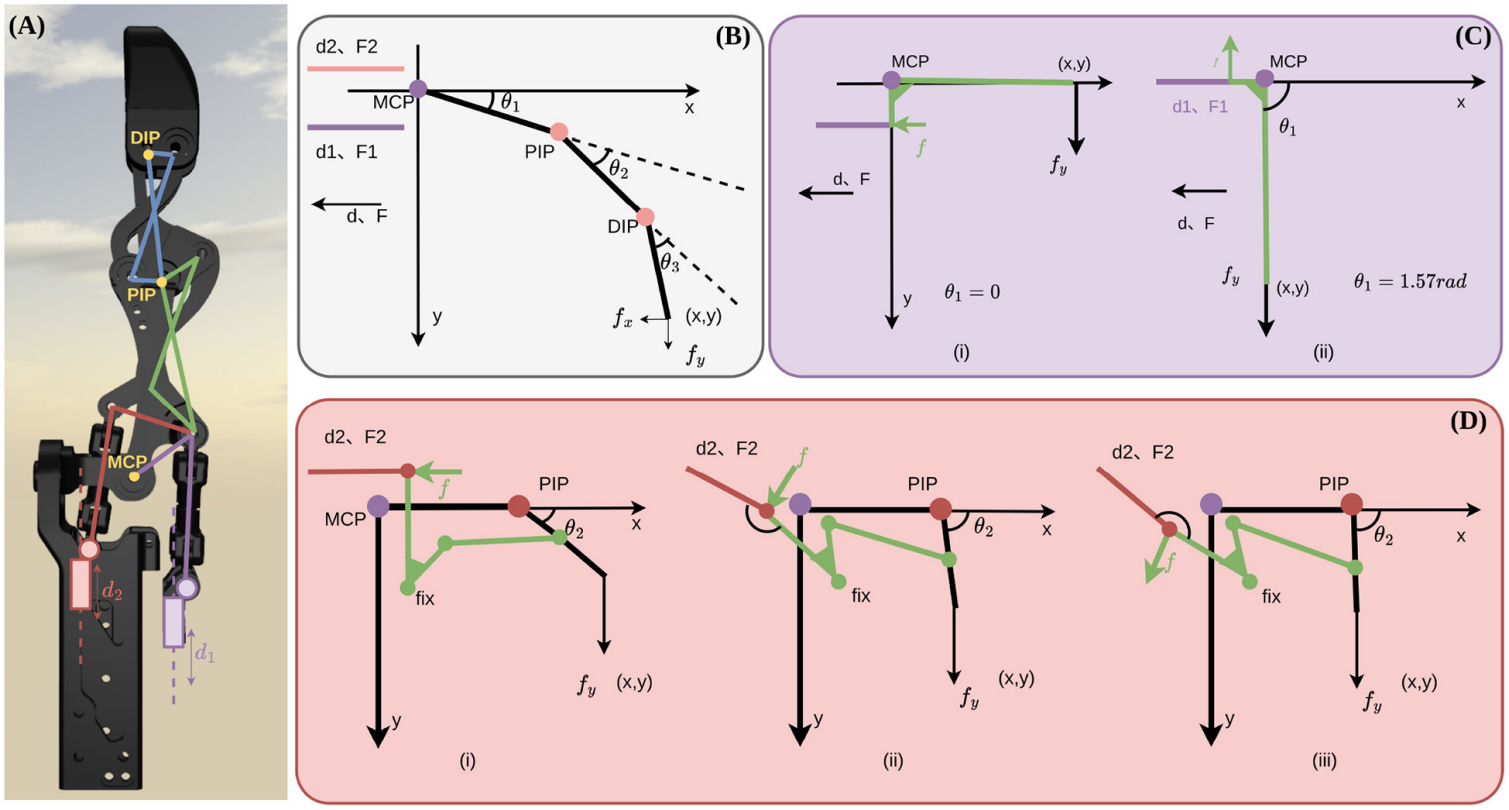
Simplified Jacobian mapping and force transmission in the tendon-linkage-driven robotic finger. **(A)** Finger link structure; MCP and ABD controlled by the first two rods, PIP by the third rod. **(B)** Three-link planar model mapping fingertip (*x, y*) to joint angles (θ_1_, θ_2_, θ_3_), then to actuator positions. **(C)** MCP link forces under external force *f*_*y*_: (i) small MCP angle: force component along rod decreases with angle; (ii) large MCP angle: force becomes perpendicular to rod, component zero. **(D)** PIP link forces under different PIP angles: (i) small angle: force along rod decreases; (ii) intermediate: perpendicular, zero component; (iii) large: component reverses direction.

As illustrated in Figure 9A, the finger is actuated by three linear pushrods: the first two rods control the MCP and ABD joints, while the third rod (denoted as *d*_2_) drives the PIP joint. To determine the actuator specifications necessary for expressive piano playing, it is essential to establish a precise mapping between the fingertip task space and the linear actuator space.

#### 3.2.1 Simplified Jacobian mapping

To facilitate the force analysis, we simplified the complex spatial linkage into a 2D equivalent model, as shown in Figure 9B. Neglecting the lateral ABD DOF for the primary striking force analysis, we define the pushrod displacement vector as $\text{d}={\left[d_{1},d_{2}\right]}^{T}$ and the joint angle vector as $\theta ={\left[\theta_{1},\theta_{2},\theta_{3}\right]}^{T}$, representing the MCP, PIP, and DIP flexion angles, respectively. Because the mapping between rod displacements and joint angles is governed by a multi-linkage geometry without a trivial analytical solution, we construct the transmission Jacobian *J*_*d*_ using numerical differentiation based on the inverse kinematics [as detailed in Supplementary Table S1 of (Kim et al. 2021)]:


$$\begin{array}{l} J_{d}=\left[\begin{array}{cc} \frac{\partial \theta_{1}}{\partial d_{1}} & \frac{\partial \theta_{1}}{\partial d_{3}}\\ \frac{\partial \theta_{2}}{\partial d_{1}} & \frac{\partial \theta_{2}}{\partial d_{3}} \end{array}\right], \end{array}$$ (3)

The fingertip position (*x, y*) is modeled as a three-link serial chain. By differentiating the forward kinematics with respect to the joint angles, we obtain the velocity Jacobian *J*_θ_. The composite Jacobian mapping the rod velocities to the fingertip velocities is then expressed as *J* = *J*_θ_*J*_*d*_. Under quasi-static conditions, the principle of virtual work and the conservation of power dictate the relationship between the actuator force F and the fingertip output force **f**:


$$\begin{array}{l} \text{F}=J^{T}\text{f}. \end{array}$$ (4)

#### 3.2.2 Numerical force analysis and configuration effects

**Figure 10:**
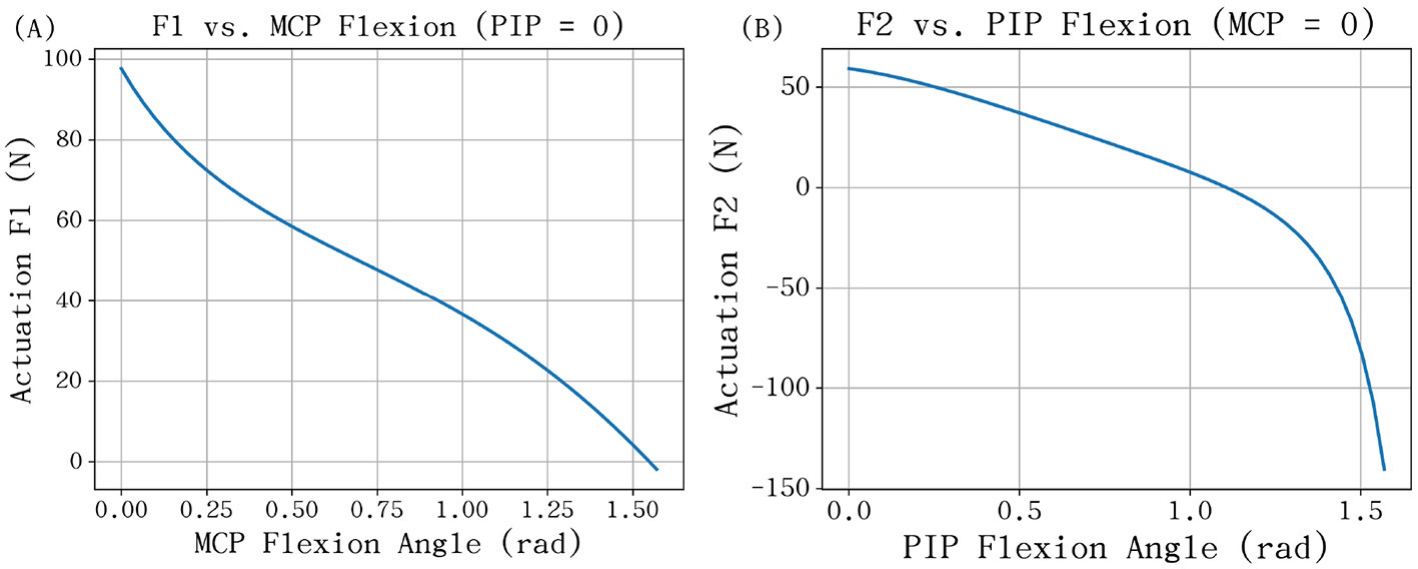
**(A)** MCP rod force *F*_1_ vs. MCP angle (PIP fixed at 0). **(B)** PIP rod force *F*_2_ vs. PIP angle (MCP fixed at 0).

Based on empirical data from professional piano performance, we evaluated the transmission model under a standard vertical fingertip load of **f** = [0, 10]^*T*^ N. The upper bound was chosen to represent a typical forte (f) dynamic level, which corresponds to a maximum key-force of approximately 9.6 N (Furuya et al., 2010). The resulting rod forces are plotted in Figure 10. For the MCP joint (Figure 10A), the required pushrod force reaches its maximum when the finger is near full extension (θ_1_≈0) and monotonically decreases as the flexion angle increases. This behavior is explained by the geometric relationship shown in Figure 9C: at low flexion angles, the linkage force vector aligns closely with the pushrod axis, maximizing the force component; as the joint flexes, the angle between the force vector and the rod increases, significantly reducing the required actuator input.

In contrast, the PIP pushrod force *F*_2_ exhibits a highly nonlinear characteristic (Figure 10B). As θ_2_ increases, the rod force initially decreases toward zero at approximately 1.1 rad, which is a singular configuration where the rod becomes perpendicular to the linkage force vector, causing the mechanical advantage to vanish (Figure 9D-i-ii). Beyond this point, the force direction reverses, requiring the actuator to resist significant back-loading (Figure 9D-iii). While this reverse-loading zone (θ_2_>1.4 rad) represents a potential risk for structural fatigue, it is rarely encountered in standard piano fingering and can be effectively avoided through software constraints.

Numerical simulations provide a rigorous basis for actuator dimensioning across the finger's workspace. For the MCP joint, the peak demand occurs at full extension, requiring a combined thrust of 100 N to produce a 10 N fingertip output. Conversely, the PIP joint force profile is highly nonlinear; the required thrust starts at 50 N and vanishes near the singular configuration (θ_2_≈1.1 rad). Although back-loading occurs at extreme flexion (θ_2_>1.4 rad), these configurations are rare in piano performance and can be avoided via control constraints, thus they are not primary drivers for motor selection.

In summary, achieving a 10 N fingertip output within the typical performance range requires a peak thrust of approximately 50 N per actuator. This establishes a 1:5 force magnification ratio between the fingertip and the transmission input, providing a definitive quantitative baseline for motor torque evaluation and final actuator specification.

### 3.3 Actuator selection and performance verification

Based on the derived velocity and force constraints, we performed a reverse-calculation to select the optimal driving units.

To ensure the robotic system can replicate the rapid transients of human motion, we first calculated the required motor speed based on the peak kinematics analyzed in Section 3.1. The MCP joint requires a peak angular velocity of ${\overset{•}{\theta }}_{\text{MCP}}^{\max}=20$ rad/s. Given the geometric relationship where a rod stroke of 18mm corresponds to a motion range of π/2, the equivalent stroke-to-angle mapping is approximately 11.5 mm/rad. Consequently, the maximum linear rod velocity is derived as:


$$\begin{array}{l} {ṡ}_{\max}=\frac{\text{d}s}{\text{d}\theta_{\text{MCP}}}\cdot {\dot{\theta }}_{\text{MCP}}^{\max}\approx 11.5\times 20=0.23\text{m}/\text{s}. \end{array}$$ (5)

With a drive shaft radius *r* = 2mm, the required motor speed is approximately 1,100 rpm:


$$\begin{array}{l} n_{m}=\frac{\omega_{m}}{2\pi }\times 60=\frac{{ṡ}_{\max}}{2\pi r}\times 60\approx 1.1\times 10^{3}\text{rpm}. \end{array}$$ (6)

The peak rod force of 50N translates to a required motor torque:


$$\begin{array}{l} \tau_{m}^{\max}=F_{\text{rod}}^{\max}\cdot r=50\times 0.002=0.10\text{N}\cdot \text{m}. \end{array}$$ (7)

The MS3506 DC servo motor was selected as the primary actuator for the system. To achieve higher rotational speeds essential for rapid finger movements, the motor driver was specifically customized. As presented in Table 4, the enhanced motor's rated speed (2, 321rpm) and peak torque (0.13N·m) fully satisfy the design requirements with a sufficient safety margin.

**Table 4:** Specifications of the enhanced MS3506 motor.

**Parameter**	**Value**
Rated torque	0.05N·m
Max torque	0.13N·m
Rated speed	2, 321rpm
Rated voltage	12V
Rated current	0.79A
Shaft diameter	4mm

By integrating human-centric dynamic analysis with analytical force transmission modeling, this chapter ensures that the proposed dexterous hand possesses the physical capacity to replicate the speed and intensity of professional piano performance.

## 4 Experimental results and performance verification

In this section, we conduct a series of experiments to evaluate the physical performance of the developed dexterous hand. The validation process focuses on three key dimensions: the biomimetic accuracy of joint coupling, the dynamic response bandwidth, and the fingertip force output capacity. These metrics collectively determine the robot's ability to execute high-speed, expressive piano performances.

### 4.1 Hand architecture and joint coupling validation

**Figure 11:**
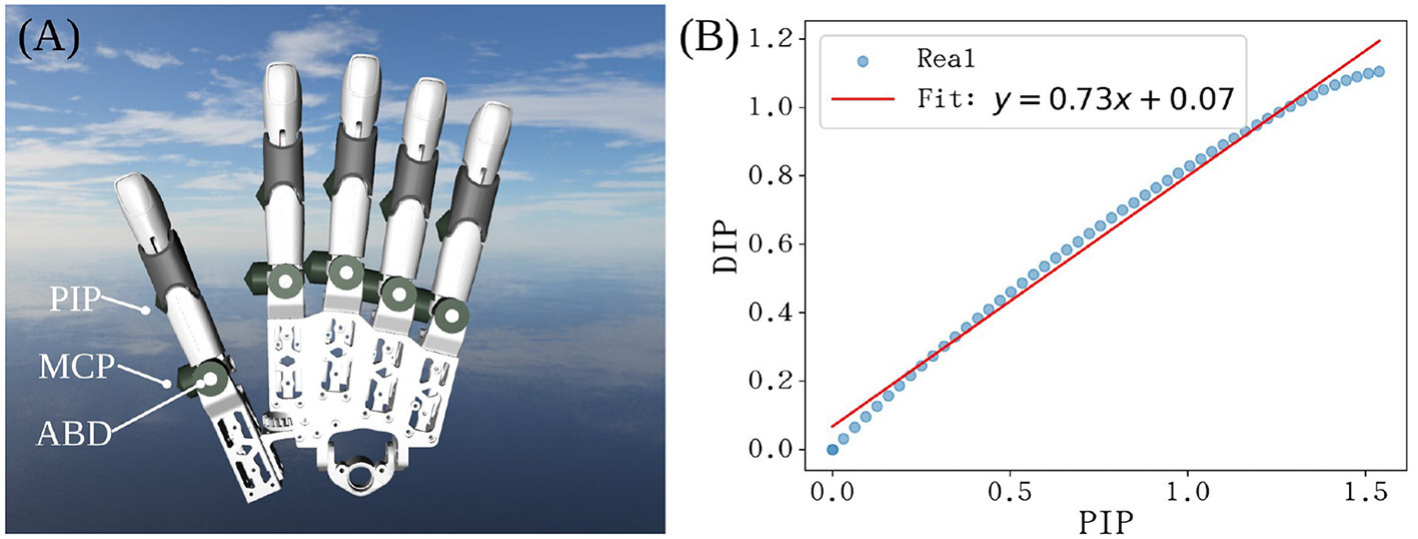
**(A)** Structural overview and active joints; **(B**) Linear relationship between PIP and DIP joint angles (*k* = 0.73) during a flexion cycle.

The developed robotic hand features a modular design where each finger integrates three active degrees of freedom (DOFs)—MCP flexion, PIP flexion, and ABD (abduction/adduction)—and one coupled DOF (DIP flexion). To replicate the natural synergy of a human hand, the DIP joint is driven by a mechanical linkage system coupled with the PIP joint (Figure 11A).

As analyzed in Section 2.4, human piano playing exhibits a strong linear correlation between PIP and DIP joints. Based on our inverse kinematics model, we calculated the geometric coupling relationship of the mechanical finger. As shown in Figure 11B, the DIP angle θ_DIP_ follows the PIP angle θ_PIP_ with a near-linear distribution across the entire workspace. The linear fit yields:


$$\begin{array}{l} \theta_{\text{DIP}}\approx 0.73,\theta_{\text{PIP}}+0.07 \end{array}$$ (8)

This coupling ratio (*k* = 0.73) highly aligns with the statistical coefficient derived from human pianist data (*k*≈0.7), demonstrating that the linkage mechanism successfully embeds human-like morphological intelligence into the hardware.

### 4.2 Dynamic velocity and system bandwidth verification

To evaluate the high-speed tracking performance and verify the system's operational bandwidth for rapid musical execution, we conducted frequency sweep experiments on the three active degrees of freedom: the MCP, PIP, and ABD joints. Sinusoidal position commands, beginning at a base frequency of 15 Hz, were applied, and the resulting trajectories were captured via high-resolution encoders.

**Figure 12:**
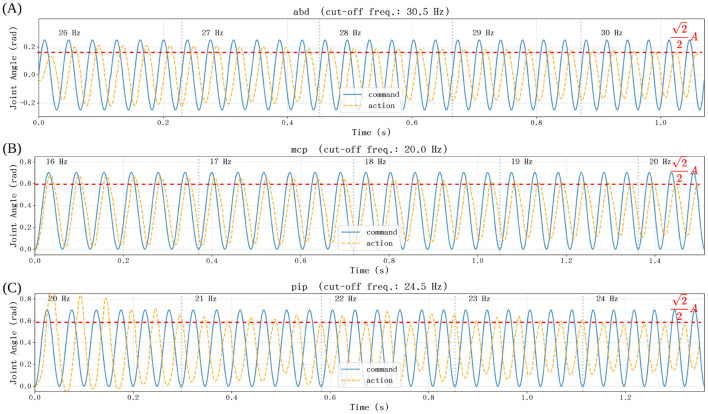
Frequency response of the robotic joints under sinusoidal position commands. The plots illustrate the angular velocity amplitude and phase lag across a frequency sweep for the **(A)** ABD, **(B)** MCP, and **(C)** PIP joints.

The experimental results, illustrated in Figures 12A–C, characterize the dynamic response of the robotic finger (Supplementary Video S1). The system's bandwidth is defined by the cut-off frequency where the response amplitude attenuates to $\frac{\sqrt{2}}{2}$ (approximately −3dB) of the command magnitude. The recorded performance metrics are as follows:

- ABD joint: achieves a bandwidth of 30.50 Hz with a peak angular velocity of 47.91 rad/s.
- MCP joint: achieves a bandwidth of 20.00 Hz with a peak angular velocity of 43.98 rad/s.
- PIP joint: achieves a bandwidth of 24.50 Hz with a peak angular velocity of 53.88 rad/s.

These results demonstrate that the hardware possesses an effective bandwidth exceeding 20 Hz. Furthermore, the peak velocities substantially exceed the biological limits of professional pianists. Even under exoskeleton-assisted training designed to surpass human motor expertise, the maximum finger movement rate is capped at 4 Hz for flexion-extension cycles (Furuya et al., 2025). For a full-stroke trajectory of 1.57 rad (90°), a 4 Hz sinusoidal motion yields a peak angular velocity of approximately 19.72 rad/s ($\omega_{peak}=A\cdot 2\pi f,A=\frac{1.57}{2}rad,f=4Hz$). Our system's peak velocity of 53.88 rad/s is significantly higher than this human-assisted ceiling, ensuring ample dynamic headroom for reproducing the most rapid and expressive musical transients.

### 4.3 Fingertip force and stiffness verification

The force output capacity of the finger is highly pose-dependent. To identify the optimal configuration for keystrokes, we utilized a three-link planar model to analyze the relationship between joint configuration, equivalent stiffness, and torque efficiency (see Supplementary Table S1 for parameters used in the simulation).

**Figure 13:**
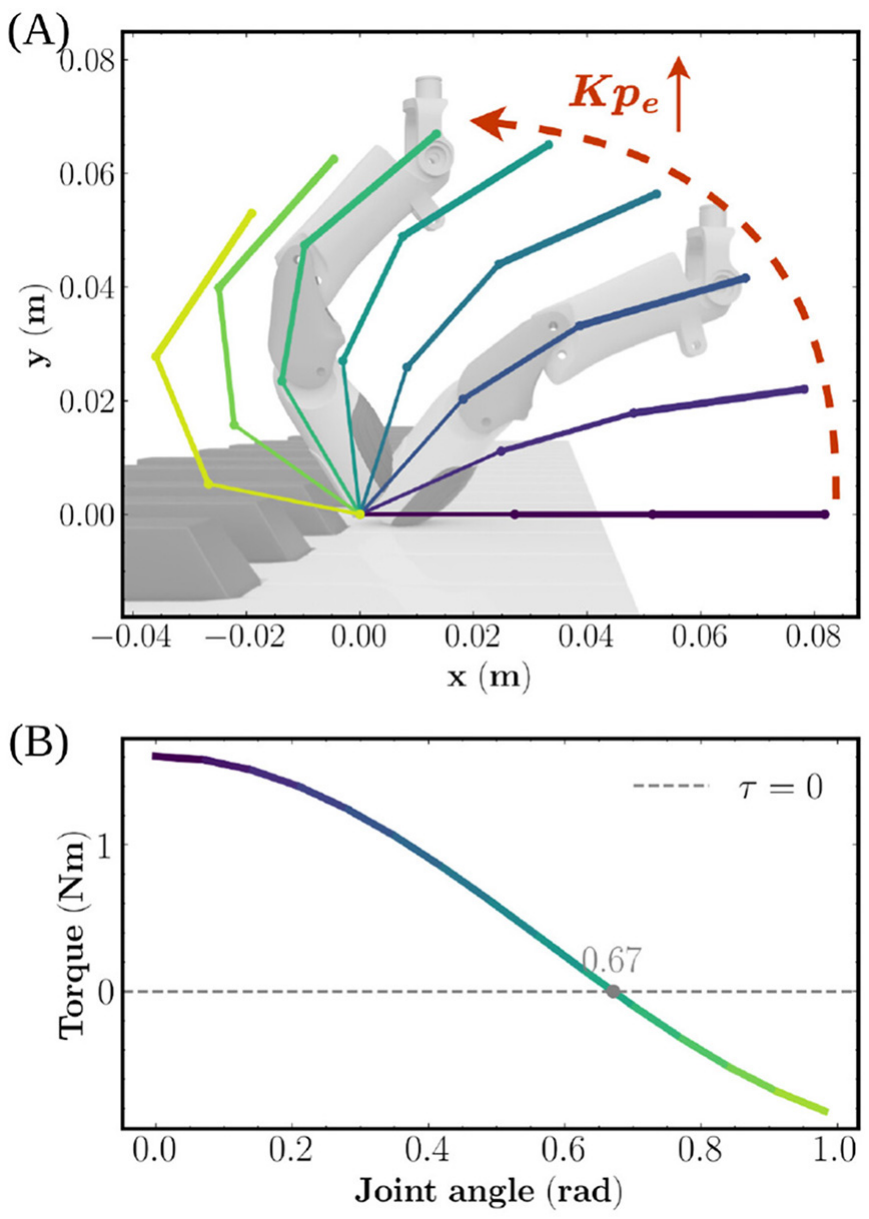
Analytical mapping of fingertip mechanics. **(A)** Equivalent vertical stiffness $K_{e}^{p}$ as a function of synchronized joint flexion; **(B)** Total joint torque required for a constant 10 N load, indicating that the most energy-efficient striking pose occurs at *q*≈0.67 rad.

Our theoretical analysis (Figure 13) indicates that the equivalent fingertip stiffness $K_{e}^{p}$ in the vertical direction increases as the finger flexes. Concurrently, the total joint torque required to sustain a 10 N load decreases significantly, reaching a minimum near a synchronized flexion angle of *q*≈0.67 rad. This configuration minimizes the moment arm between the MCP joint and the fingertip, maximizing force transmission efficiency.

**Figure 14:**
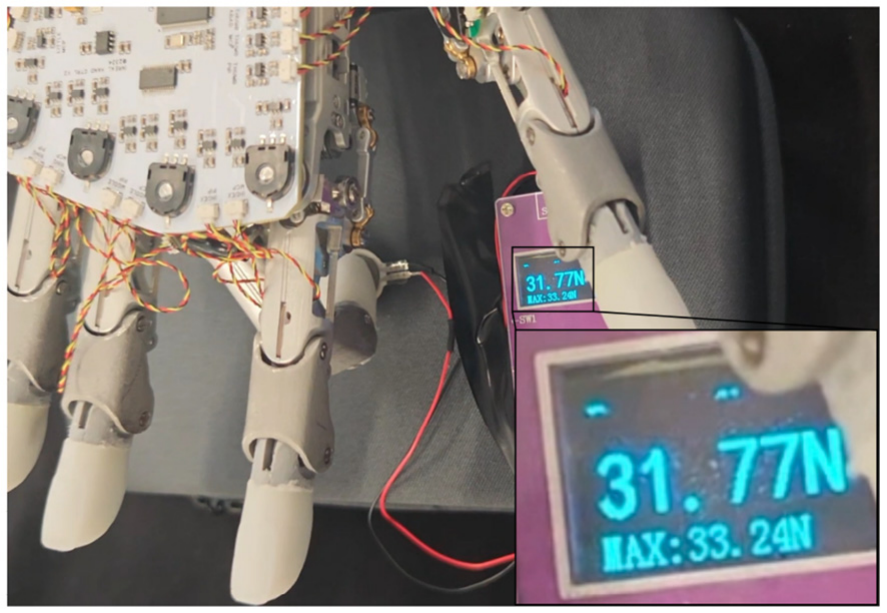
Fingertip maximum output force experiment. The robotic finger was adjusted to the model-predicted optimal configuration (*q*_1_ = *q*_2_≈0.67 rad) to perform vertical pressing against a force sensor. The measured maximum output force reached an upper limit of 33.24 N.

In the physical validation, the finger was moved to this optimal pose to press against a force sensor. As shown in Figure 14, the robotic hand achieved a maximum fingertip force of 33.24 N (Supplementary Video S2). Although this output remains below the 50 N peaks observed in extreme fortissimo staccato transients, it substantially exceeds the 15 N threshold typical of mezzo-forte staccato. Given that legato playing requires even lower force levels due to its smoother, sustained contact, a peak of 33.24 N provides sufficient dynamic range to replicate the majority of professional piano repertoires (Askenfelt and Jansson, 1991, 1992).

### 4.4 Piano performance

To evaluate the practical performance of the proposed dexterous hand in complex musical tasks, a human-robot collaborative experiment was conducted using a reinforcement learning-based motion controller. As demonstrated in Supplementary Video S3, the robotic hand was tasked with performing the main melody of a Grade 7 difficulty piece *Croatian Rhapsody*, while a human pianist provided harmonic accompaniment. This setup requires the hardware to maintain high-speed finger transitions and precise dynamic control to ensure temporal synchronization with the human performer.

**Figure 15:**
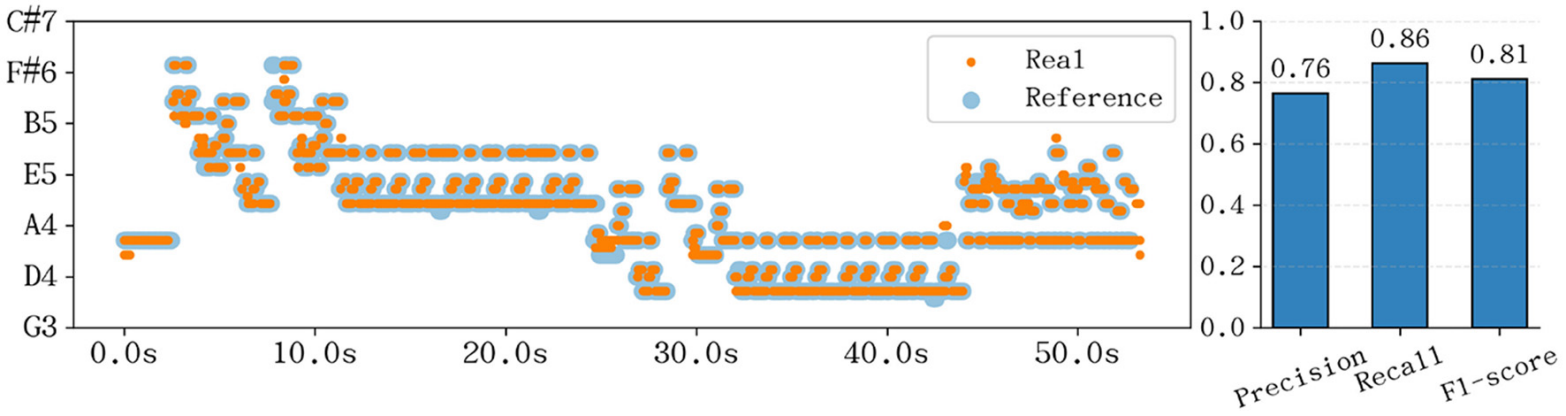
**(Left)** Piano roll comparison between the reference score (blue) and the real-world performance (orange), showing high temporal and pitch alignment. **(Right)** Performance metrics including Precision, Recall, and F1-score for both simulation and real-world experiments.

The performance accuracy was quantitatively assessed using the F1-score, defined as the harmonic mean of precision and recall:


$$\begin{array}{l} F_{1}=2\cdot \frac{\text{Precision}\cdot \text{Recall}}{\text{Precision}+\text{Recall}} \end{array}$$ (9)

where a correct note is defined by matching both pitch and onset time. As shown in Figure 15, the robotic hand achieved an F1-score of 0.81. This result validates that the mechanical design provides sufficient bandwidth and stability to execute professional-level repertoire with high fidelity.

## 5 Conclusion

This study presents a bio-inspired, high-performance robotic hand framework optimized for the rigorous demands of expressive piano performance. By embedding human-centric kinematic synergies—notably the stable *k*≈0.7 PIP-DIP coupling ratio—directly into a linkage-driven architecture, we successfully bridged the “actuation gap” to achieve human-like dexterity with significantly reduced control complexity. Experimental validation confirms that the developed prototype fulfills virtuoso-level requirements, demonstrating a peak angular velocity of 53.88rad/s, a 30Hz dynamic bandwidth, and a maximum fingertip force of 33.24N, thereby matching or exceeding human physiological limits for nuanced musical modulation.

Beyond the domain of robotic music, this research offers a scalable paradigm for designing specialized end-effectors in diverse high-impact, high-speed manipulation scenarios. Future efforts will focus on refining lateral kinematics through a specialized thumb module with a customized *k* = 1.85 ratio and integrating multi-modal tactile feedback to enable adaptive, closed-loop touch control.

## Data Availability

The raw data supporting the conclusions of this article will be made available by the authors, without undue reservation.
