# Supplementary material for: Design an anthropomorphic dexterous hand for expressive piano performance
Source: Front Neurorobot. 2026 Apr 15;20:1775834. doi: 10.3389/fnbot.2026.1775834 (PMC13125105; doi:10.3389/fnbot.2026.1775834)
Supplement: Supplementary file 4 [file Data_Sheet_1.pdf]

Supplementary Material

1 SUPPLEMENTARY FIGURES

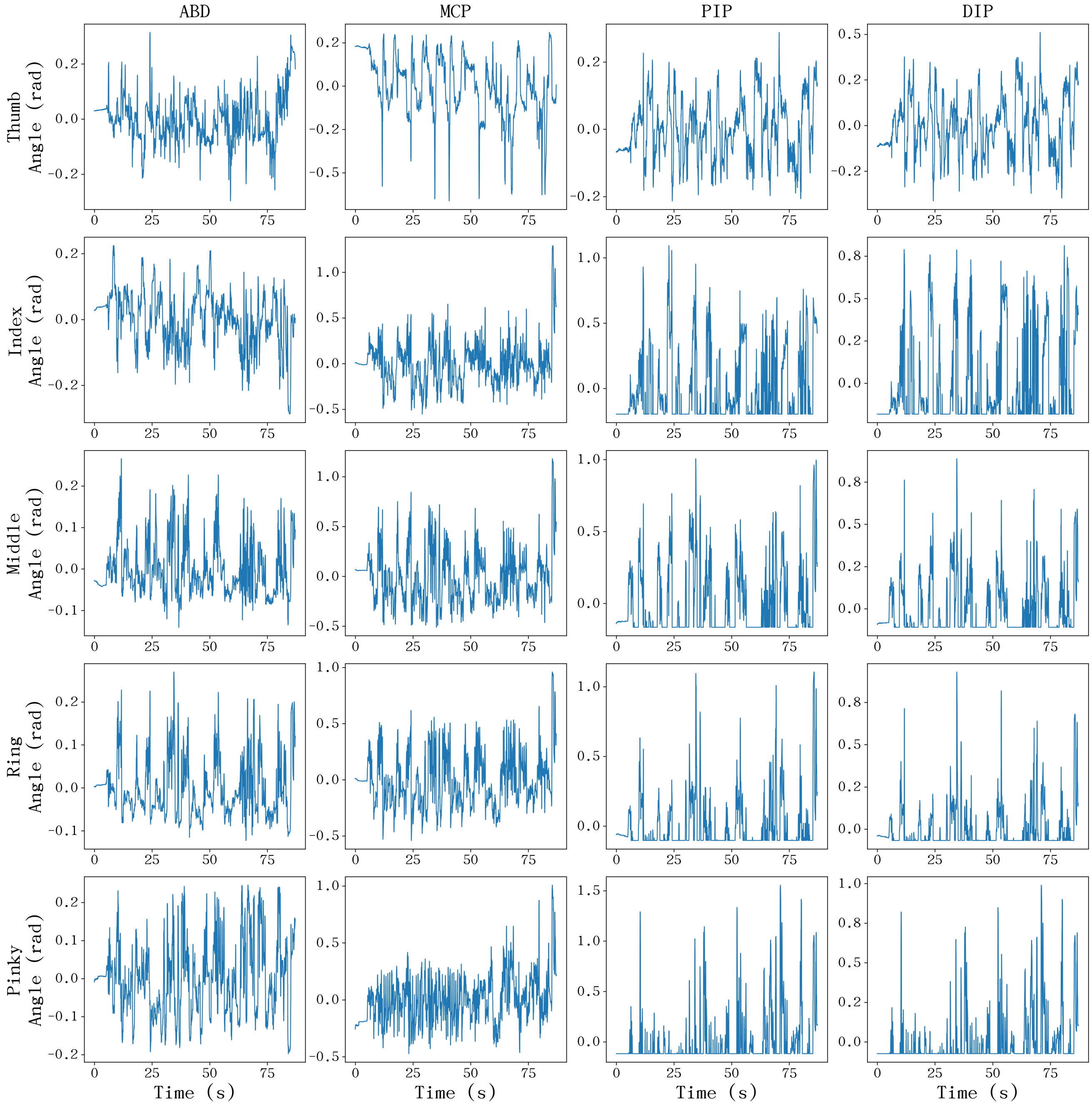

Figure S1. Time-domain trajectories of finger joint angles during piano playing. Rows correspond to fingers and columns correspond to joints, providing the temporal context for the frequency-domain analysis.

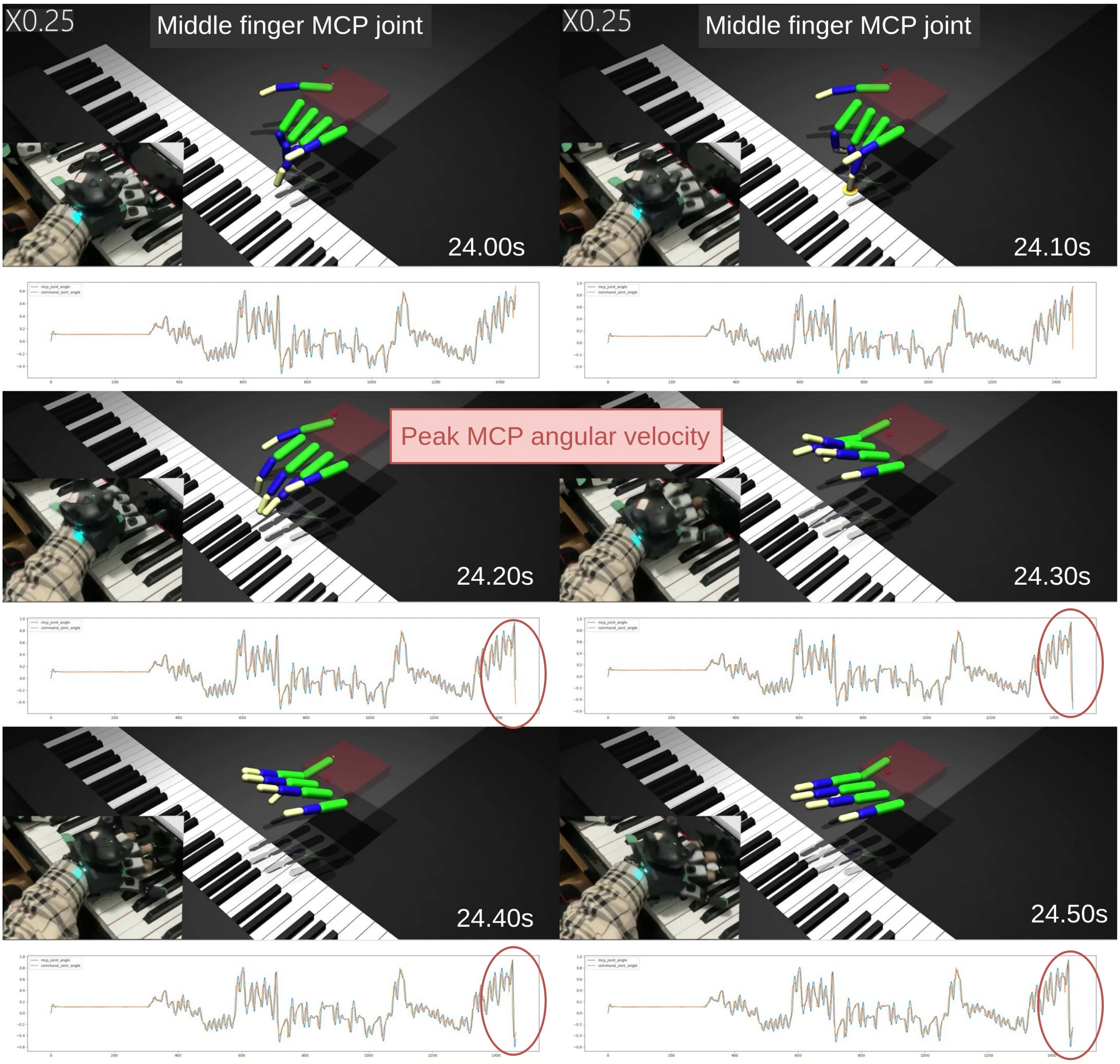

Figure S2. Detailed visualization of a representative peak MCP angular velocity event around 24 s. Six frames spanning 24.00 s to 24.50 s show slow-motion (0.25× speed) video snapshots of the human pianist (bottom-left of each subfigure), corresponding MuJoCo simulations of an anatomically consistent hand model, and the associated joint angle trajectories. The two central frames correspond to the instant of peak MCP angular velocity, revealing a rapid transition from a flexed, key-pressing posture to an extended state during finger release.

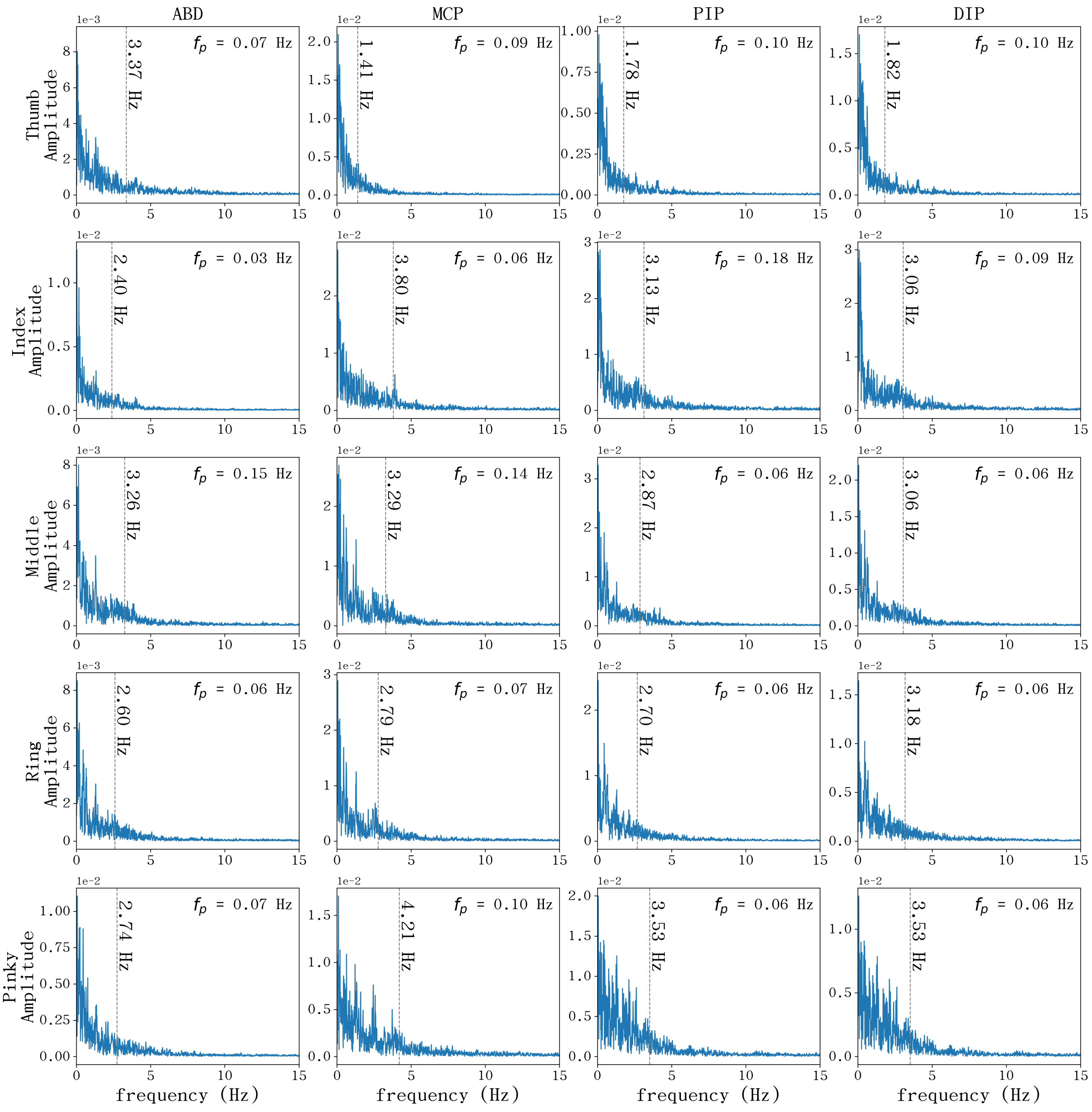

Figure S3. Frequency-domain analysis of finger joint angles during piano playing. In each subplot, f_p denotes the peak frequency, while the dashed vertical line indicates the cutoff frequency containing 95% of the signal energy.

2 SUPPLEMENTARY TABLES

Table S1. Finger link parameters used in the three-link model. Each link's length l_i, mass m_i, and joint stiffness k_i are listed below.

Link	Length l_i (m)	Mass m_i (kg)	Joint Stiffness k_i (Nm/rad)
Proximal	0.0303	0.020	10
Middle	0.0243	0.015	10
Distal	0.0272	0.010	10
